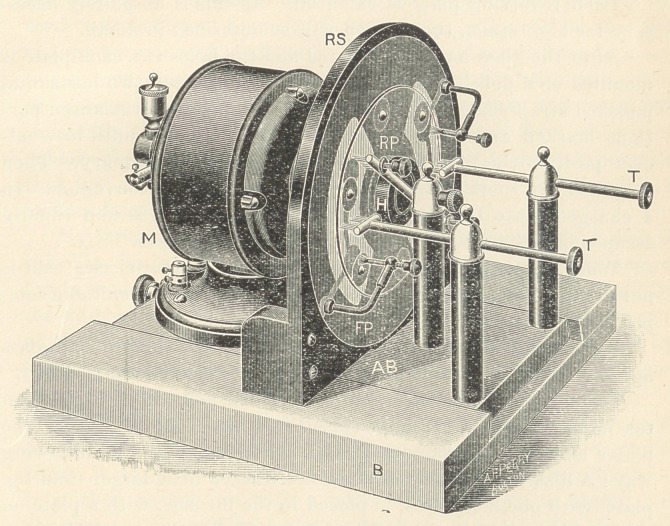# Directly Connected Static Machines

**Published:** 1896-10

**Authors:** William Rollins

**Affiliations:** Boston, Mass.


### DIRECTLY CONNECTED STATIC MACHINES.

BY WILLIAM ROLLINS, BOSTON, MASS.

SINCE Röntgen's discovery static machines have assumed increased importance, because tubes excited by them give fine definition, which is of great importance, particularly in dental work.

As the voltage of static machines is not dependent on the size of the plates or on their number, it is practical to use small plates, obtaining the required quantity of electricity by increasing the speed of rotation. In applying this plan to the machines in the market several serious obstacles are met, chief among which are vibration and noise. These defects I overcome in the machines, one type of which is here figured. Select a shunt-wound motor and place a variable resistance in the armature circuit so the speed can be regulated. Mount on an adjustable sliding support, *AB*, held in grooves on the base *B*, and movable by means of the milled head. This arrangement allows the distance between the plates *FP* and *RP*, to be changed. On the end of the armature-shaft is a hard-rubber hub, *H*, carrying the revolving glass plate *RP*, on the surface of which may be the ordinary metal buttons, or the surface may be entirely without metal, or the buttons may be attached in an improved way to be hereafter described. *RS* is a hard-rubber support which is recessed for the fixed plate *FP*, and serves also to support the fixed brushes. This means of support was applied to the machine by Mr. Zeigler, and while it certainly protects the glass plate, I am not sure that the insulation is as perfect as with the ordinary form of support. Boring holes through the fixed plate to receive the arms which support the diagonal brushes (which is an invention of Philip Atkinson) is certainly a marked improvement on the ordinary method. The terminals, *TT*, are hollow to receive the ends of the conducting wires, which are securely held by the chucks shown.

Every part of the machine should be insulated, and the edges of the plates rounded to prevent loss into the air.

Each revolving plate is balanced. As this is absolutely necessary for high speed, the method will be described in detail.

After the glass has been selected and the holes cut, each plate is mounted on a polished steel axis and placed between two horizontal polished strait edges. It is allowed to turn, the overbalanced portions marked, and the corresponding edges ground, until in whatever position the plate is placed it will remain stationary. Then the edges are rounded in order to prevent loss by convexion. In a charged plate the electricity collects at the edges and rapidly escapes into the air from all rough edges and corners.

When properly balanced, the plates are never true circles, as it is not practical to obtain glass of an even thickness of a suitable composition.

I grind the edges of the plates instead of the sides, for all glass appears to have a skin, which, if removed, weakens the material.

When a very powerful machine is needed, and plates larger than ten inches are used (at high speed) it is necessary to flatten the plates as well as to balance them. This is done in the following way: A disk of cast iron, three inches thick, a little larger than the plate, with one face plate, is placed in the furnace, with a plate on top, and slowly heated until the glass flattens. Iron and glass are then allowed to cool twelve hours.

Where sectors or buttons are used on the plates, the ordinary means of fastening with shellac may be improved upon by using platinum in thin pieces and fusing onto the glass by enamel at the time the plates are being flattened. Such sectors do not become detached. I shall figure an improved form of Wimshurst machine in the November number, as this type seldom reverses.

*Summary of Improvement.*—Direct connection with motive power; high speed to reduce size of machine; flattened plates to avoid vibration; balanced plates to avoid vibration; rounded edges to plates to avoid loss; platinum sectors or buttons fused to plates; covering parts with rubber to avoid loss.

## Figures and Tables

**Figure f1:**